# Targeting oncogenic Myc as a strategy for cancer treatment

**DOI:** 10.1038/s41392-018-0008-7

**Published:** 2018-02-23

**Authors:** Hui Chen, Hudan Liu, Guoliang Qing

**Affiliations:** 1 Zhongnan Hospital of Wuhan University, Wuhan, People’s Republic of China; 2 Medical Research Institute, Wuhan University, Wuhan, People’s Republic of China

## Abstract

The *MYC* family oncogene is deregulated in >50% of human cancers, and this deregulation is frequently associated with poor prognosis and unfavorable patient survival. Myc has a central role in almost every aspect of the oncogenic process, orchestrating proliferation, apoptosis, differentiation, and metabolism. Although Myc inhibition would be a powerful approach for the treatment of many types of cancers, direct targeting of Myc has been a challenge for decades owing to its “undruggable” protein structure. Hence, alternatives to Myc blockade have been widely explored to achieve desirable anti-tumor effects, including Myc/Max complex disruption, *MYC* transcription and/or translation inhibition, and Myc destabilization as well as the synthetic lethality associated with Myc overexpression. In this review, we summarize the latest advances in targeting oncogenic Myc, particularly for cancer therapeutic purposes.

## Cancer: Therapeutic strategies target myc from all directions

The cancer gene myc is the subject of a variety of research strategies aimed at developing anti-cancer therapies. Myc, which regulates at least 15% of all genes including those involved in cell development and survival, is mutated and overexpressed in many cancer types. While inhibiting myc is an obvious anti-cancer strategy, directly targeting myc has proved difficult. Dr Guoliang Qing and colleagues from Wuhan University have reviewed advances in myc-targeting strategies as potential cancer therapies. Current strategies include indirectly targeting myc by inhibiting its regulation and stability, and interaction with its partner Max, which is necessary for its regulation of gene expression. Other strategies involve inducing a mutation in a gene that interacts with myc to kill cancer cells. These and future innovative approaches provide promise for effective therapeutics against myc-dependent cancers.

## Introduction

**Fig. 1:**
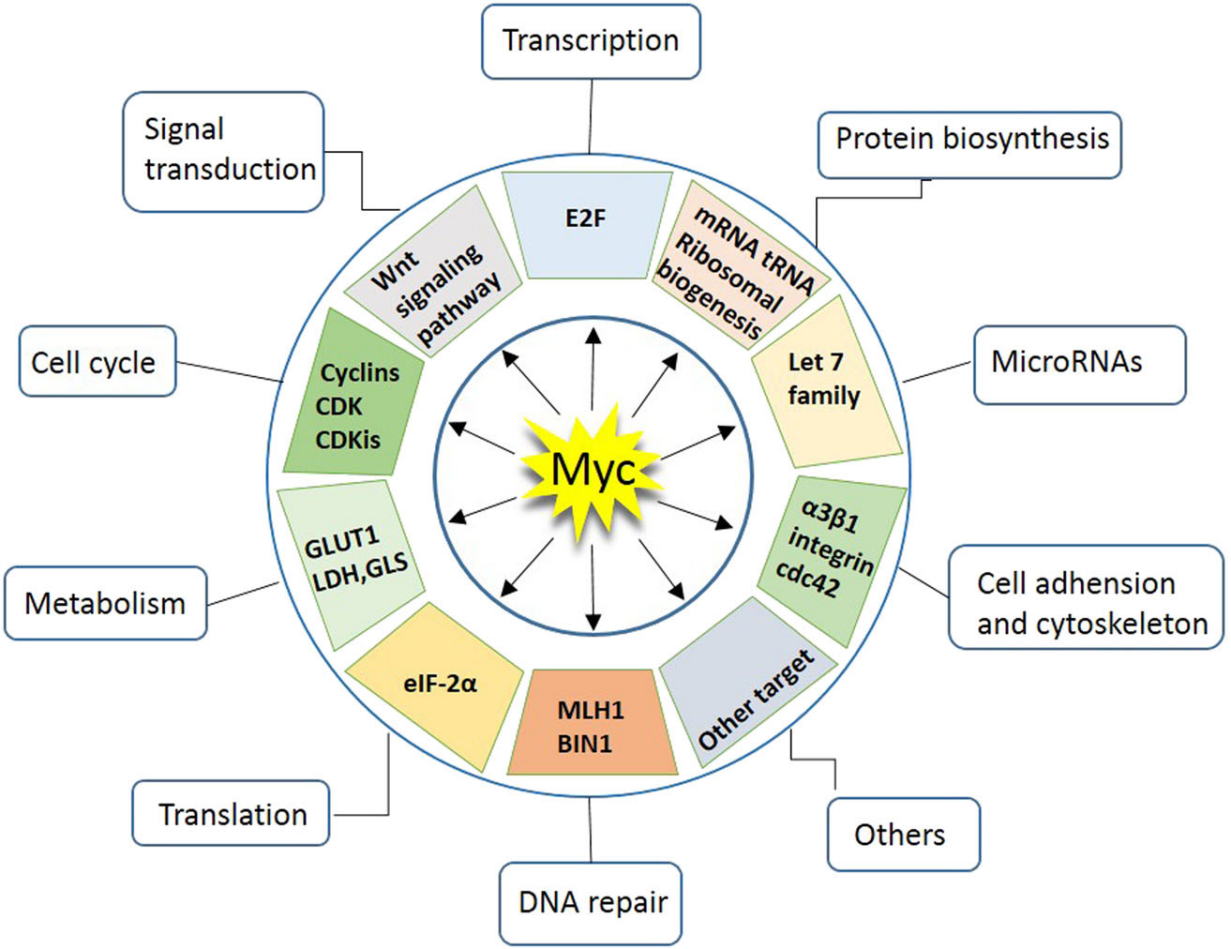
Myc regulates a spectrum of cellular functions. Myc regulates a large number of protein-coding or non-coding genes that are involved in distinct cellular functions, including cell cycle, protein biogenesis, cell adhesion, metabolism, signal transduction, transcription, and translation, among others

The *MYC* oncogene family consists of three members, *C-MYC*, *MYCN*, and *MYCL*, which encode c-Myc, N-Myc, and L-Myc, respectively.^1–3^ The Myc oncoproteins belong to a family of so-called “super-transcription factors” that potentially regulate the transcription of at least 15% of the entire genome.^4^ The major downstream effectors of Myc include those involved in ribosome biogenesis, protein translation, cell-cycle progression and metabolism, orchestrating a broad range of biological functions, such as cell proliferation, differentiation, survival, and immune surveillance (Fig. 1).^4, 5^

**Fig. 2:**
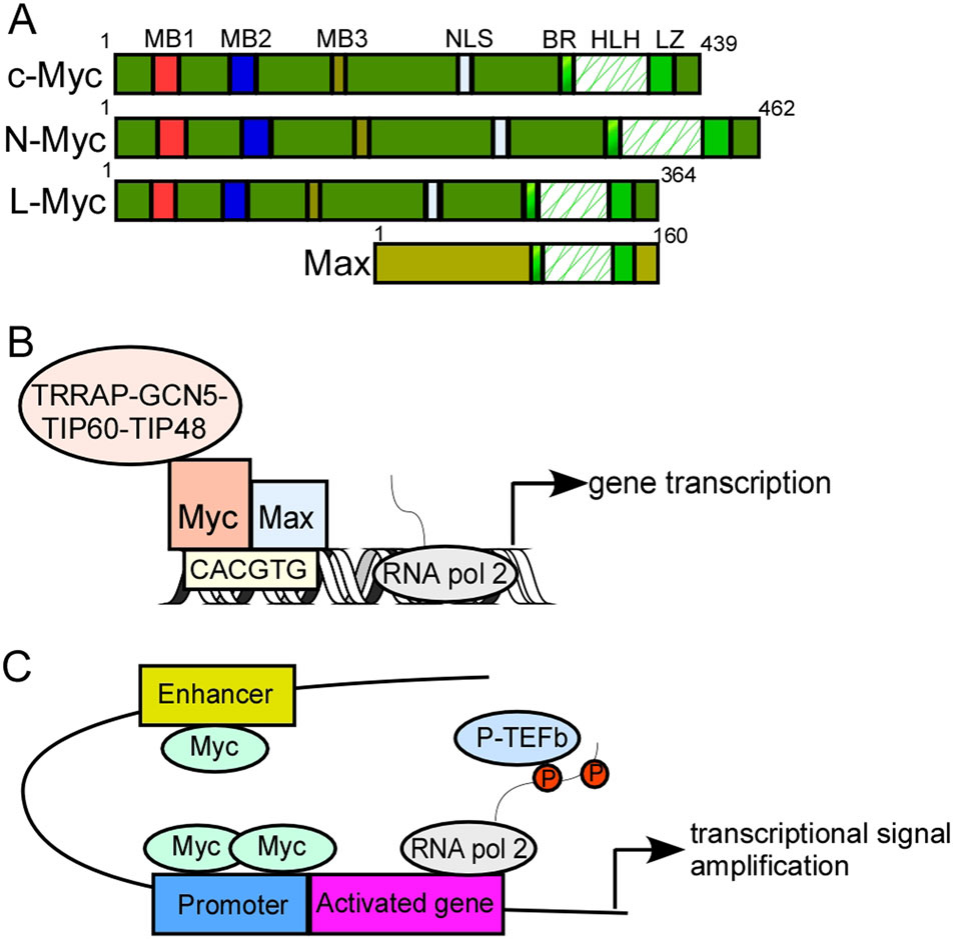
Transcriptional activation of target genes by Myc family members. **a** protein structure of Myc family members. The N terminus of Myc comprises a transactivation domain (TAD) and three highly conserved elements, known as Myc boxes 1–3. Myc box 1 (MB1) possesses a phosphodegron, which is targeted by the ubiquitin E3 ligase FBW7. MB2 is required for all the known functions of Myc and recruits a histone acetyltransferase (HAT) complex, MB3 regulates Myc protein stability and transcriptional activities. The C-terminal domain contains a basic-region /helix-loop-helix/leucine-zipper (BR/HLH/LZ) motif that is necessary for DNA–protein interactions. Max, the partner of Myc, binds with Myc through the C-terminal BR/HLH/LZ motif. **b** Myc functions as a transcription factor. Upon binding to CACGTG (E-box), the Myc–Max dimeric complex recruits chromatin-modifying complexes, including GCN5, TIP60, TIP48, and TRRAP, leading to transcriptional activation. GCN5 and TIP60 are histone acetyltransferases; TIP48 is an ATP-binding protein, TRRAP transactivation/transformation-associated protein. **c **Myc functions as a transcriptional signal amplifier. In this model, Myc binding is not E-box dependent. Myc accumulates in the promoter and enhancer region of all active genes and causes transcriptional signal amplification

Myc family members exhibit high-structural homology, including the basic-region /helix-loop-helix/leucine-zipper (BR/HLH/LZ) motif at the C terminus and three highly conserved elements, known as Myc boxes 1–3 at the N terminus (Fig. 2a).^1, 2, 5^ As a master transcription factor, Myc binds to Max through the common BR/HLH/LZ motif, which is required for DNA–protein interactions.^1, 2, 5^ The Myc/Max heterodimer recruits a chromatin-modifying complex (TRRAP, GCN5, TIP60, and TIP48) and activates transcription by binding to the conserved E-box DNA sequence (CACGTG) located in the transcriptional regulatory region of target genes (Fig. 2b).^1, 2, 5^ Recent studies have shown that, in addition to recognizing specific E-box sequences, c-Myc also accumulates in the promoter regions of active genes, leading to transcriptional amplification (Fig. 2c).^6, 7^

The expression of Myc family members is tightly controlled under normal circumstances.^1, 5^ Yet, Myc is frequently deregulated in human cancers. Excess Myc expression can be induced upon retroviral promoter insertion, chromosomal translocation/amplification, activation of super-enhancers within the *MYC* gene, and/or mutation of upstream signaling pathways that enhance Myc stability.^5^ Studies in transgenic mouse models have demonstrated that even transient inactivation of Myc elicits tumor regression, suggesting that regulation of oncogenic Myc could be harnessed to treat cancer patients.^8–10^ Yet, drug development aimed at directly targeting Myc has proved challenging. First, as a transcription factor, Myc lacks a specific active site for small molecules, making it difficult to functionally inhibit its activities using strategies similar to those used for kinases. Second, Myc is predominantly located in the nucleus, thus, targeting nuclear Myc with specific monoclonal antibodies is technically impractical. To overcome these obstacles, alternative approaches to indirectly abrogate Myc oncogenic functions have been extensively investigated.

## Indirect targeting of Myc

**Fig. 3:**
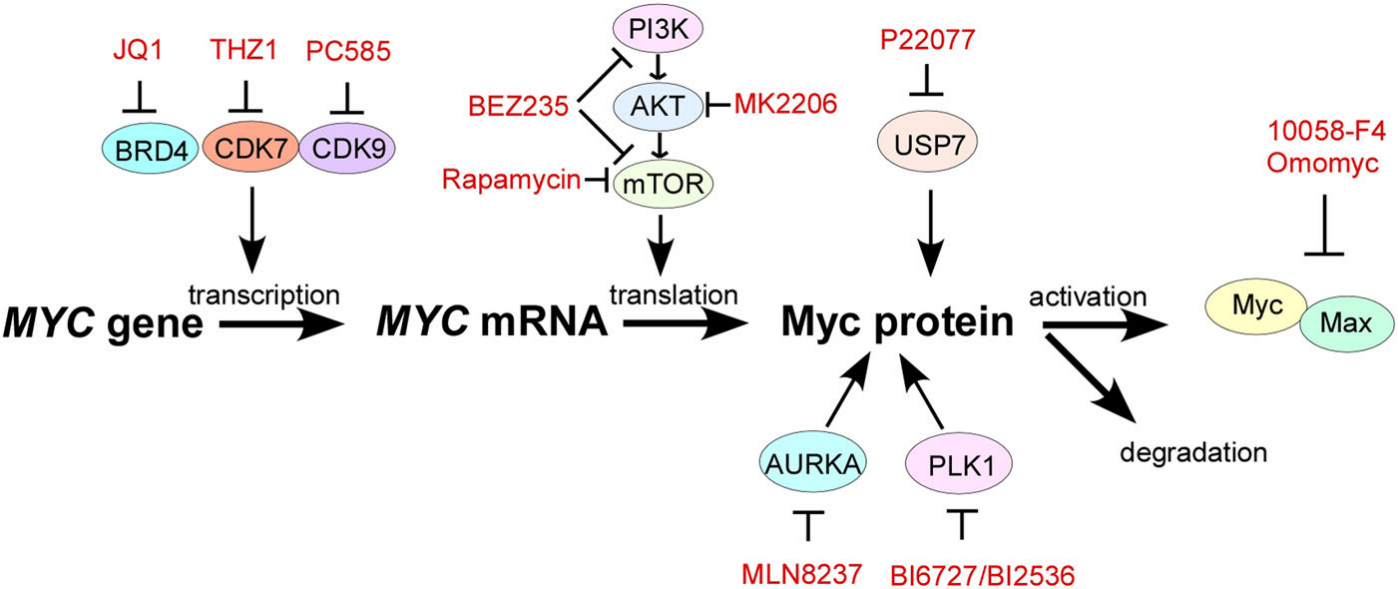
Various strategies to target Myc. Inhibitors of BRD4, CDK7, and CDK9 inhibit *MYC* expression at the transcriptional level. Inhibition of the PI3K/AKT/mTOR pathway blocks *MYC* translation, whereas USP7, AURKA, and PLK1 inhibitors destabilize Myc at the posttranslational level. 10058-F4 and Omomyc function to interrupt the Myc–Max dimeric complex. BRD4 bromodomain-containing 4, CDK7 cyclin-dependent kinase 7, CDK9 cyclin-dependent kinase 9, PLK1 polo-like kinases 1, PI3K/AKT/mTOR phosphatidylinositol 3-kinase/AKT/mammalian target of rapamycin

Because strategies to directly target Myc have not been achieved thus far, essential targets involved in Myc deregulation have been exploited as new approaches to treat Myc-driven cancers. Targeting *MYC* transcription by interfering with chromatin-dependent signal transduction to RNA polymerase, a process in which BRD4 has been implicated, has shown great promise.^11, 12^ Myc stability is tightly controlled by the ubiquitin-proteasome system, thus, a potential strategy to target Myc is to selectively inhibit the kinases and/or deubiquitinases that stabilize Myc.^13, 14^ Myc strictly depends on its partner Max to regulate gene transcription, so interrupting the Myc–Max complex is therefore an additional approach to inhibit Myc signaling.^15^ Here, we provide a concise overview of the key factors involved in the transcription, translation, stability, and activation of Myc, which could be targeted for the treatment of Myc-addicted cancers (Fig. 3).

## Targeting *MYC* transcription

### Bromodomain-containing 4 (BRD4)

BRD4 is a member of the mammalian bromodomain and extraterminal (BET) family.^16^ BRD4 regulates transcription through recruitment of the positive transcription elongation factor b (P-TEFb), which phosphorylates the carboxy-terminal domain of RNA polymerase II (pol II), to the site of hyperacetylated chromatin.^17^ These changes lead to the release of RNA pol II from pausing in the promoter-proximal region, ultimately resulting in transcriptional elongation.^17, 18^ *MYC* transcription is under BRD4 regulation. JQ1, a powerful inhibitor of BRD4, competes with BRD4 for binding to acetylated lysines and displaces BRD4 from the super-enhancers within the *MYC* oncogene.^11, 12^ As such, inhibition of the BET bromodomain with JQ1 showed potent anti-cancer effects both in vitro and in vivo in multiple hematopoietic cancers and pancreatic ductal adenocarcinoma (PDAC) exhibiting *C-MYC* overexpression.^19–22^ Neuroblastomas and other *MYCN*-driven cancers are also sensitive to BET inhibitors.^23^ GSK525762, a specific BET inhibitor, is currently in early-phase clinical trials for treating these hematopoietic malignancies and solid tumors (ClinicalTrials.gov: NCT01943851, NCT03266159).

### Cyclin-dependent kinase 7 and 9 (CDK7 and CDK9)

In contrast to the classical cell-cycle CDKs which are largely responsible for cell-cycle transition, CDK7 and CDK9 are CDKs that have critical roles in transcription initiation and elongation.^18, 24^ CDK7 is a catalytic subunit of the transcription factor IIH complex (TFIIH), and CDK9 is a kinase subunit of P-TEFb.^25, 26^ These two transcriptional kinases phosphorylate specific serine residues within the carboxy-terminal domain of Pol II, facilitating efficient transcriptional initiation, pause release and elongation.^27^ Numerous studies demonstrate that inhibition of transcriptional CDKs primarily affects the accumulation of transcripts critical for the control of cell identity, growth, and proliferation.^28–30^

A general feature of *MYC* deregulation is its transcriptional regulation by Super-Enhancers (SEs), clusters of enhancers that are densely occupied by transcription factors and chromatin regulators, including CDK7 and CDK9, rendering this group of kinases ideal candidates for blocking Myc-dependent transcriptional amplification.^30, 31^ Indeed, inhibition of CDK7 and/or CDK9 substantially reduces *MYC* expression, attendant to widespread transcriptional downregulation of Myc target genes.^30, 32, 33^ Administration of specific inhibitors against CDK7 (THZ1) and/or CDK9 (PC585) induced potent anti-tumor effects in *MYC*-overexpressing T-cell acute lymphoblastic leukemia, mixed-lineage leukemia, neuroblastomas, and small cell lung cancers, validating these newly developed transcriptional CDK inhibitors as a potential treatment strategy that targets global transcriptional amplification in Myc-driven cancers.^30, 32, 33^

## Targeting *MYC* mRNA translation

### Mammalian target of rapamycin (mTOR)

The phosphatidylinositol 3-kinase (PI3K)/AKT/mTOR pathway is frequently altered in various cancers.^34^ mTOR is a serine/threonine kinase that functions as the catalytic subunit of two distinct complexes called mTOR complexes 1 and 2 (mTORC1 and mTORC2).^35^ The central role of mTOR in protein synthesis is largely attributed to mTORC1.^35, 36^ mTORC1-dependent phosphorylation of eukaryotic translation initiation factor 4E (eIF4E) binding protein 1 (4EBP1) blocks its ability to negatively regulate the translation initiation factor eIF4E, thus promoting the translation of mRNAs containing long 5′-untranslated regions (5′-UTRs) with complex RNA secondary structures, such as *MYC*.^35, 36^ As such, pharmacological inhibition of the PI3K/AKT/mTOR pathway markedly decreased Myc level and exhibited remarkable therapeutic efficacy in Myc-driven cancers, including neuroblastoma, small-cell lung carcinoma, breast cancer, and multiple hematopoietic cancers.^34, 37–39^

### Cytoplasmic polyadenylation element-binding protein (CPEB)

The CPEB-family proteins are sequence-specific RNA-binding proteins which control the elongation of the poly(A) tail and polyadenylation-induced translation.^40^ CPEB binds the cytoplasmic polyadenylation element (CPE) containing the conserved UUUUAU or UUUUAAU sequence within the 3′-UTRs of responding mRNAs.^40^ A recent study revealed that the *C-MYC* mRNA contains CPEs that can be recognized by CPEB.^41^ Mechanistically, CPEB recruits Caf1 deadenylase through an interaction with Tob, an antiproliferative protein, and inhibits c-Myc expression by accelerating the deadenylation and decay of its mRNA.^42^ Expression of CPEB-family proteins are frequently downregulated in human cancers.^40^ Therefore, pharmacological approaches aimed at reactivating CPEB expression would lead to Myc inhibition in Myc-driven cancers.

## Targeting MYC stability

### USP28, USP36, and USP7

Myc stability is tightly controlled by the ubiquitin-proteasome system.^1^ Upon phosphorylation at Thr58, Myc is polyubiquitinated by the E3 ligase FBW7 and degraded by the proteasome.^43^ The human FBW7 locus encodes three protein isoforms, FBW7α, FBW7β, and FBW7ɣ, that differ in their N-terminal sequences and in their subcellular localization.^44^ Both FBW7α and FBW7ɣ are responsible for the selective degradation of endogenous Myc in human cells.^44^ Several deubiquitinating enzymes are involved in Myc stabilization. USP28 was shown to bind c-Myc through an interaction with FBW7α and antagonize its E3 ligase activities in the nucleus, leading to Myc stabilization and tumor cell proliferation.^45^ USP36 deubiquitinates and stabilizes c-Myc through interactions with FBW7ɣ in the nucleolus.^46^ USP7 directly binds to and stabilizes N-Myc through deubiquitination in neuroblastomas cells, and a small-molecule inhibitor of USP7, P22077, markedly suppressed growth of *MYCN*-amplified neuroblastoma in a xenograft model.^13^ In principle, targeting these deubiquitinases could cause Myc destabilization and tumor suppression.

### AURKA

The Aurora family includes AURKA, AURKB, and AURKC, which are key regulators of mitosis.^47^ AURKA contributes to tumorigenesis through interactions with P53 and Myc.^48–50^ Recently, Otto et al.^14^ showed that Aurora A and N-Myc acted as oncogenic partners in neuroblastomas. AURKA forms a complex with N-Myc, which protects N-Myc from FBW7-mediated proteasomal degradation.^14^ Two AURKA inhibitors, MLN8054 and MLN8237, disrupt the Myc–AURKA complex, resulting in N-Myc degradation and tumor regression in *MYCN*-amplified neuroblastomas.^14, 51^ MLN8237 also induced c-Myc degradation in P53-mutant human hepatocellular carcinoma cells.^52^ These data suggest that AURKA inhibitors may be potential therapeutics for the treatment of Myc-dependent cancers.

### Polo-like kinase 1 (PLK1)

The Polo-like kinases (PLKs) comprise a family of five serine/threonine protein kinases that control many crucial biological processes.^53^ The best characterized PLK family member is PLK1. Using *MYCN*-amplified neuroblastomas and small cell lung carcinomas as model systems, we recently demonstrated that PLK1 and Myc created a positive, feedforward activation loop that was essential for sustaining mutual high expression, leading to Myc-dependent transcriptional amplification and aggressive tumor progression. Inhibitors of PLK1, such as BI 6727 or BI2356, preferentially induce potent apoptosis of Myc-overexpressing tumor cells and synergistically potentiate the therapeutic efficacies of BCL-2 antagonists. These findings reveal a PLK1-FBW7-Myc signaling circuit that underlies tumorigenesis and validate PLK1 inhibitors, alone or with BCL-2 antagonists, as potential effective therapeutics for Myc-overexpressing cancers.^54^

## Targeting the MYC–MAX complex

The Myc–Max complex is required for the binding of Myc to DNA and its subsequent activation of target gene transcription.^1^ The Myc–Max dimer interface is a parallel, left-handed, four-helix bundle, with each monomer comprising two R-helices separated by a loop.^55^ Although studies have shown that this structure has no apparent sites for positioning a small-molecule inhibitor, several labs have screened small molecules that block this interaction. The peptide mimetic IIA6B17 was first reported as a small-molecule inhibitor of Myc–Max dimerization.^15^ A compound called 10058-F4 was capable of disrupting the Myc–Max complex in HL60 cells.^56^ Another widely known inhibitor, Omomyc, a mutant basic helix-loop-helix peptide that sequesters Myc in a transcriptionally incompetent complex, prevents Myc-induced tumorigenesis in multiple mouse tumor models.^57–59^

## Synthetic lethal interaction with MYC

**Fig. 4:**
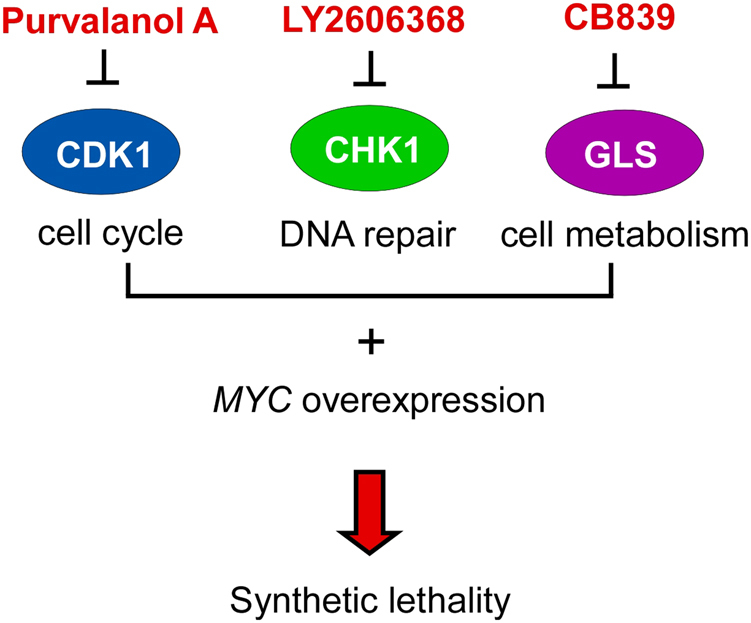
Synthetic lethal interactions with Myc deregulation. Myc-mediated synthetic lethality has been observed with various targets, including CDK1, CHK1, and GLS. CDK1 cyclin-dependent kinase 1, CHK1 checkpoint kinase 1, GLS glutaminase

Two genes (“A” and “B”) are said to be “synthetic lethal” if mutation of either gene alone is compatible with viability but simultaneous mutation of both genes causes death.^60^ Synthetic lethal interactions are most commonly described for loss-of-function alleles but can also involve gain-of-function alleles.^60^ For example, gene A might become essential for survival when a particular gene B is overexpressed. This situation describes Myc-mediated synthetic lethality. *MYC* overexpression is found in many cancers, and *MYC* overexpression sensitizes cells to apoptosis, enabling targeting a gene that is synthetic lethal to a cancer-relevant *MYC* overexpression should kill only cancer cells but spare normal counterparts. Here, we describe key factors that exhibit synthetic lethal interactions with Myc deregulation (Fig. 4).

### Cyclin-dependent kinase 1 (CDK1)

CDK1 is a catalytic subunit of the highly conserved protein kinase complex known as the M-phase-promoting factor, which is essential for the G1/S and G2/M-phase transitions of the eukaryotic cell cycle.^61^ RNAi screens for synthetic lethality in *MYC* overexpressing cells highlight the promise of targeting this cell-cycle kinase for Myc-dependent cancers.^62^ Indeed, inhibiting CDK1 function using the small molecule purvalanol A selectively induced apoptosis in cells with *MYC* overexpression and significantly decreased tumor growth in Myc-dependent lymphoma and hepatoblastoma mouse models.^63^ It appears that the selective induction of apoptosis upon CDK1 inhibition is associated with upregulation of the pro-apoptotic molecule BIM and/or downregulation of the anti-apoptotic molecule survivin.^62, 63^ It should be noted that CDK1 inhibition could also selectively kill transformed cells by targeting E2F-1 and/or enhancer of zeste homolog 2 (EZH2).^64, 65^ Most likely, multiple mechanisms contributed to the CDK1 inhibition-induced tumor regression. Nevertheless, these results suggest the potential value of targeting CDK1 in Myc-driven cancers.

### Checkpoint kinase 1 (CHK1)

CHK1 has a key role in cell-cycle progression and DNA damage checkpoint control.^66, 67^ The use of CHK1 inhibitors to treat cancer was derived from observations that tumor cells without DNA damage checkpoints during tumorigenesis or therapy are highly sensitive to additional genomic instability.^68^ Myc deregulation is sufficient to induce genome instability.^69^ Myc induces replication stresses and DNA damages through excessive replication-fork firing, making Myc-overexpressing tumors substantially more sensitive to CHK1 inhibition.^69^ As such, CHK1 inhibition leads to massive cell death in Myc-overexpressing lymphomas, neuroblastomas, breast and lung cancers.^70–72^

### Glutaminase (GLS)

Many tumor cells rely on glutamine metabolism to fuel their unabated growth and proliferation.^73, 74^ Oncogenic Myc increases the surface expression of glutamine transporters and alters mitochondrial metabolism, making the cell dependent on exogenous glutamine for survival.^75–79^ Glutamine is converted to glutamate by GLS, an enzyme that is highly expressed in tumor cells. Accordingly, inhibition of glutamine metabolism by GLS inhibitors selectively induces apoptosis in Myc-overexpressing tumor cells.^75–80^ Of note, CB-839, a potent and selective GLS inhibitor, is currently in phase I clinical studies for treating leukemias and other hematological tumors with Myc deregulation.^81^

## Conclusion and perspectives

Here, we have described multiple pharmacological approaches to indirectly target Myc at different levels (Table 1). These approaches should be translated as a strategy to move forward in future patient care, as patients with Myc deregulation are likely to respond. Although direct targeting of Myc has not yet been achieved, promise remains in developing innovative approaches to effectively and specifically target this cancer super-controller. As a matter of fact, BCL-2 was also considered undruggable until a decade of fragment-based nuclear magnetic resonance (NMR) screening altered and broadened the view of this potential inhibitory molecule.^82^ Whether through direct or indirect targeting of Myc, better therapeutics to target Myc-dependent cancers will be required in the future.

**Table 1:** Small moleculars linked to Myc-pathway inhibition

Target	Compound names	Clinical testing	References
*MYC* transcription			
BRD4	JQ1	Preclinical testing only	11,12
	GSK525762	Phase1/2 in solid and hematologic malignancies	83,84
CDK7	THZ1	Preclinical testing only	30,32
CDK7/CDK9	Roscovitine	Phase 1/2 in advanced solid tumors	85
CDK9	Flavopiridol	Phase 1/2 in hematologic malignancies	28, 29,86
	PC585	Preclinical testing only	33
*MYC* translation			
mTORC1	Rapamycin/RAD001/CCI-779	Phase 1/2/3/4 in multiple cancers	37, 87,88
AKT	MK2206	Phase 1/2 in multiple cancers	89,90
PI3K/mTOR	BEZ235	Phase 1/2 in multiple cancers	38,88
Myc stabilization			
USP7	P22077	Preclinical testing only	13
USP28	Not available		45
USP36	Not available		46
AURKA	MLN8237	Phase 1/2 in multiple cancers	14,51
PLK1	BI 6727	Phase 1/2/3 in advanced solid tumors and AML	91,92
	BI 2536	Phase 1/2 in advanced solid tumors and AML	93,94
Myc activation			
Myc–Max complex	10058-F4	Preclinical testing only	56
Synthetic lethality			
CDK1	Purvalanol A	Preclinical testing only	63
	P276-00	Phase 1/2 in multiple cancers	95
CHK1	LY2606368	Phase 1/2 in multiple cancers	96,97
GLS	CB-839	Phase 1/2 in solid and hematologic malignancies	80,81
